# Diabetes Self-Management Education: Miles to Go

**DOI:** 10.1155/2013/581012

**Published:** 2013-03-20

**Authors:** Helen Altman Klein, Sarah M. Jackson, Kenley Street, James C. Whitacre, Gary Klein

**Affiliations:** ^1^Division of Research, MacroCognition LLC, P.O. Box 533, Yellow Springs, OH 45387, USA; ^2^Department of Psychology, Wright State University, Dayton, OH 45435, USA

## Abstract

This meta-analysis assessed how successfully Diabetes Self-Management Education (DSME) interventions help people with type 2 diabetes achieve and maintain healthy blood glucose levels. We included 52 DSME programs with 9,631 participants that reported post-intervention A1c levels in randomized controlled trials. The training conditions resulted in significant reductions in A1c levels compared to control conditions. However, the impact of intervention was modest shifting of only 7.23% more participants from diabetic to pre-diabetic or normal status, relative to the control condition. Most intervention participants did not achieve healthy A1c levels. Further, few DSME studies assessed long-term maintenance of A1c gains. Past trends suggest that gains are difficult to sustain over time. Our results suggested that interventions delivered by nurses were more successful than those delivered by non-nursing personnel. We suggest that DSME programs might do better by going beyond procedural interventions. Most DSME programs relied heavily on rules and procedures to guide decisions about diet, exercise, and weight loss. Future DSME may need to include cognitive self-monitoring, diagnosis, and planning skills to help patients detect anomalies, identify possible causes, generate corrective action, and avoid future barriers to maintaining healthy A1c levels. Finally, comprehensive descriptions of DSME programs would advance future efforts.

## 1. Introduction

Diabetes afflicts approximately 25.8 million people in the United States, or 8.3% of the population. Type 2 diabetes, or non-insulin dependent diabetes mellitus (NIDDM), accounts for 90 to 95% of all diagnosed cases of diabetes in adults [1]. The problem is growing, particularly among young people. Over their lifetime, patients experience increased risks of complications including blindness, kidney damage and failure, cardiovascular disease, nerve damage, and lower-limb amputation. A NIH study from 2011 estimated that costs related to the treatment of diabetes accounts for about $174 billion of the national health care annually [1]. 

Type 2 diabetes complications stem from the inability of the body to use insulin properly, resulting in heightened blood glucose levels [2]. This is measured with the HbA1c test, the percent of glycated hemoglobin in the blood. It is commonly called the A1c. The NIH criterion for diagnosing diabetes is an A1c of 6.5 or higher although this varies somewhat with the individual. Pre-diabetes is between 5.7 and 6.4. A reading below 5.7 is normal [3].

The U.K. Prospective Diabetes Study (UKPDS) [4] found that over a ten-year period, each 1% reduction in A1c (e.g., from 8.5% to 7.5%) was associated with reductions of risk of 21% for death, 14% for myocardial infarctions, and 37% for microvascular complications. They found no threshold value for risk mitigation confirming the value A1c reductions. Research studies, however, rarely follow patients for even a few years so clinical thresholds remain the best assessment of treatment impact. 

This research addresses the question of how effectively current DSME interventions help patients with type 2 diabetes achieve sustained control of their blood glucose. Physicians and other health care professionals can prescribe effective medications, provide optimal dietary guidelines, and support needed life style modifications. In the end, however, it is the patient and their caregivers who must make the daily decisions needed to control blood glucose.

There is a wide array of support strategies for patients. Web sites maintained by The National Institute of Health (NIH) [5], the American Diabetes Association (ADA) [6], and the American Association of Diabetes Educators (AADE) [7] provide state-of-the-art recommendations and online support. These include guidelines for food choices and the timing of meals, exercise, and responses to stress and illness. Pharmacology research has produced new and better medications. Human factors practitioners have improved glucose measurement devices [8] and have developed reminder systems to support adherence [9].

With the growing burden of diabetes on health care systems and the plethora of medical, pharmaceutical, and human factors advancements, it is critical that DSME programs increase their effectiveness, sustainability, and scalability. This meta-analysis of interventions started with six reviews of DSME [10–15]. The interventions differed in sponsorship, duration, target group, and educational approach. Three used meta-analyses [10–12]; one adopted a systematic review procedure [13]; one used both meta-analysis and systematic review procedures [14]; and one used a data mining procedure [15]. Each assessed the impact of DSME interventions on glycemic control, a critical barometer of self-management success.

Ellis et al. [10] surveyed 28 studies with a total of 2,439 participants and found A1c reductions of 0.32% for intervention groups compared to control groups. Gary et al. [11] examined 18 studies with a total of 2,720 participants, and found a significant pooled effect size (standardized mean difference) of −0.43 in A1c. Norris et al. [12] examined 31 studies with 4,263 participants, and found an average GHb reduction, a measure related to glycemic control, of 0.76% post intervention.

Ismail et al. [14] reviewed 12 studies with 1,390 participants and reported a pooled standardized difference of −0.32 in the A1c between experimental and control conditions. Sigurdardottir et al. [15] looked at 18 studies with 4,293 participants. They found that only four of the eighteen interventions attained a post-intervention. A1c level of 6.4% or less, the boundary between diabetes and pre-diabetes used by some researchers. The current accepted value for healthy A1c is 5.7% or lower. 

While these reviews reported modest but statistically significant reductions in A1c levels among intervention participants when compared to control participants, most reductions did not reach healthy A1c levels. Intervention participants remained at risk from elevated blood glucose levels as reflected by A1c or other standards metrics. Finally,Norris et al. [13] provide a classification of 72 studies from 84 articles. Each is described by intervention type and outcome variables. It provides a useful summary of research, but not a quantitative analysis of outcomes. 

More disheartening in these reviews, reductions in glycemic control were often not sustained over time. The meta-analysis conducted by Norris et al. [13] illustrates this trend: studies with a post-intervention follow-up period of six months or less showed greater effectiveness in glycemic control than those with longer follow-up periods. Only 12 of the 72 programs reviewed by Norris et al. [13] had follow-ups of 1 year or later and only two of these found that reductions were maintained. While the later meta-analysis by Norris et al. [12] found a 0.76 reduction in HbA1c from baseline immediately after the intervention, this improvement decreased to 0.26 several months later. 

These six overview studies encountered a variety of difficulties and limitations. First, behavioral change techniques have generally lacked standardized definitions and taxonomies [17]. DSME studies in particular do not follow standard routines for delivering care and reporting their results. Further, randomized controlled trials, basic to intervention research, were not universal. Control group treatments were often listed only as “usual care,” with contact times missing in the report [10, 12]. For example, Sigurdardottir et al. [15] report that in at least 7 of the 18 interventions studied, the control groups received more than “standard care” and also showed significant improvements. Ellis et al. [10] also reported that overall control groups showed decreases in A1c levels of 0.66 from baseline at follow-up. Norris et al. [13] found both intervention and control groups exhibited glycemic improvement in 15 of the studies included; in three of these, the control group improved more than the intervention group did. Norris et al. [12] also reported greater improvements in several of their control groups.

Another research problem has been attrition rates. Sarkisian et al. [18] noted high attrition rates in many of their studies, with one reaching 50%. Norris et al. [12] reported that attrition was greater than 20% in one-third of the studies included in their analysis. Norris et al. [13] found studies that reported significant decreases in glycated hemoglobin levels either used very intense interventions or had significantly higher attrition rates. The high attrition rates would skew the outcomes if the least successful patients dropped out. The more of these drop-outs, the better the results would appear even if the DSME program had no real benefit.

Because of these methodological problems, we share Gary et al.'s [11] conclusion that current interventions “yield improvements in glycemic control that are promising, but not yet compelling.” We conducted this meta-analysis to address the problems outlined above. We wanted to minimize the methodological problems identified in the past reviews. Our meta-analysis included DSME research studies from the six previous meta-analysis efforts that provided A1c measures both pre- and post-intervention. We also included later studies to capture more recent DSME projects [16, 19–69]. Studies were included based on their selection criteria and methodological quality as specified below. We excluded studies that only measured A1c less than 13 weeks after the start of the intervention because this interval is too short to reflect A1c changes. Thus, we attempted to compile a set of DSME studies that met more stringent criteria and allowed easier comparisons across studies. 

A second reason for our analysis was to provide an initial exploration of the dynamics of self-management. Lippa et al [70] report that rule-based instructional programs for patients with type 2 diabetes are less likely to be effective because patients often have difficulty applying a large set of rules in complex situations. Similarly, lifestyle self-management interventions for diet, weight loss, and exercise are notoriously difficult to sustain. Klein and Lippa [71] found that patients leading active lives needed cognitive self-management skills for self-monitoring, including guidance for interpreting their A1c levels and understanding the implications of their data for anticipating, preventing, and repairing problems. This meta-analysis explored current DSMEs approaches. We were particularly interested in how cognitive self-management versus rules and procedures might impact outcomes.

## 2. Materials and Methods

### 2.1. Strategy for Identifying DSME Studies

We reviewed the studies from the six earlier referenced reviews addressing the effect of DSME interventions on blood glucose control [10–15]. We supplemented these with additional studies published from 2005 through 2009 using the same strategies as Ellis et al. [10], Ismail et al. [14], and Sigurdardottir et al. [15]. The selection strategy reflected the standards for articles reporting meta-analyses in psychology [72]. We searched MEDLINE, PsycInfo, and CINAHL for English language publications using the keywords “diabetes mellitus” combined with each of the following: *“*patient education,” “educational intervention,*” “*self-management education,*”* “psychological therapies,” and “clinical trials*.”*

 The titles and abstracts of search results were assessed for relevance and retrieved if appropriate. When the same data were used in multiple publications, we included only one of the publications in our analyses.

### 2.2. Criteria for Inclusion

All of the studies in our analysis met the following criteria.

- Intervention participants completed a DSME intervention designed to increase adherence and only data collect Per Protocol (PP) was included.
- Participants were adults with type 2 diabetes as defined by NIH [3]. We excluded people with type 1 diabetes, gestational diabetes, or unspecified type of diabetes.
- A1c values were available as both baseline and post-intervention measures and data were sufficient to define the means and standard deviations for the A1c.
- All studies used randomized controlled trials meeting at least one of the following criteria:
  1. Random assignment of participants from a single pool (e.g., treatment center, unified recruitment method).
  2. Study specified as a randomized trial (unless evidence suggested otherwise, such as significantly different participant baseline characteristics).
  3. Study sites were randomly assigned and equivalent (with sufficient evidence).
  4. Groups were matched on baseline measures.

### 2.3. Criteria for Exclusions

**Figure 1:**
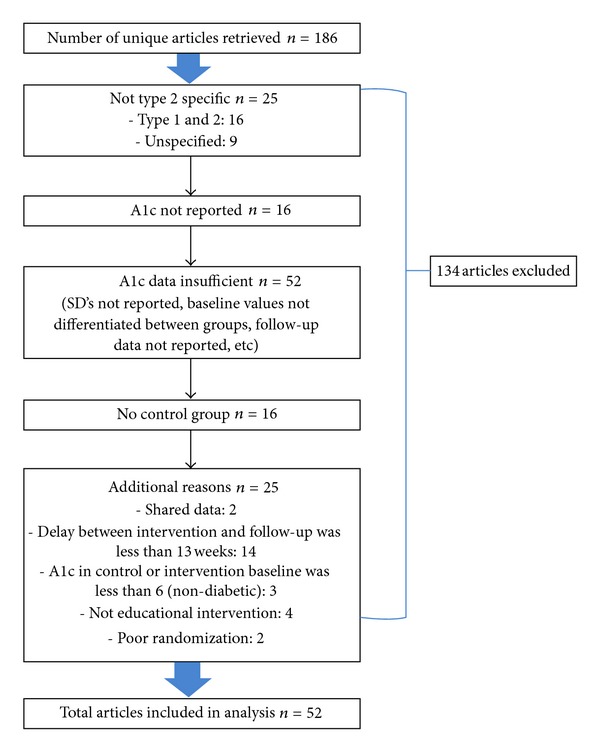
Excluded article chart.

A total of 186 unique articles were retrieved. Of these, 134 studies were excluded for the reasons provided in Figure 1. The included studies satisfied the criteria listed above. One study [73] was removed because its Cohen's *d* was more than three standard deviations above the mean, the cutoff for detecting outliers. 

Table 1 describes the 52 studies [16, 19–69], with a total of 9,631 participants included in our analyses. The studies are described by author, publication year, and sample size. Five additional parameters are noted, when available, for each study: the person delivering the intervention, the intervention content, mode of delivery, the treatment duration in weeks, and the time before follow-up in weeks. The variables of intervention profession, content, and delivery mode, are detailed in Table 1.

We classified the content based on the description provided in the program. In some cases, several content areas were mentioned.

Rules and Procedures (RP) was the most commonly mentioned content and focused on explicit guidelines, such as specific rules regarding diet and exercise. An example of a procedure would be how to perform blood glucose monitoring. Rules and procedures can include the use of a journal for recording data but do not typically provide support for translating blood glucose readings into effective decision making.

Affective and Emotion (AE) focused on emotion, motivational encouragement, empowerment, and/or confidence building.

Social and Situational (SS) focused on managing social and situational factors that impede effective diabetes self-management. These strategies might include holiday meal planning and selecting restaurant meals. 

Complex Cognition (CC) focused on mental models or other complex cognitive strategies designed to use conceptual understanding of diabetes to moderate blood glucose levels. This goes beyond the simple application of rules and procedures to the use of a mental model to detect anomalies and identify causes, and to generate corrective and preventive strategies.

## 3. Results

### 3.1. DSME Effectiveness

The overall results showed that the DSME interventions significantly reduced A1c levels (Table 2). The final mean A1c levels in the intervention group (*M* = 7.61, SD = 1.34) were lower than that of the control group (*M* = 8.18, SD = 1.43), *t*(146) = 3.51, *P* < 0.01. This is not surprising as studies without intervention effects were less likely to be submitted for publication or to be accepted if submitted. Nevertheless, it is useful to examine the effect size in preparation for more detailed examination of the factors that contributed to these effects.

The overall intervention effect was to reduce mean A1c levels from 8.70% to 7.61%, as shown in Table 2, a reduction of 1.09% that was significant at the 0.01 level. However, the control groups also reduced their A1c levels from 8.70 to 8.18, a reduction of 0.52% that was also significant at the 0.01 level. We used an analysis of variance to compare A1c gain score (post-treatment minus baseline) with treatment (intervention versus control) as the independent variable. The decrease in A1c levels was significantly greater for the intervention condition, *t*(146) = 3.51, *P* < 0.01.

To put these findings into context, we computed an “Impact Score” reflecting the proportion of participants whose A1c levels were at or below 6.4%. The NIH guidelines set 6.5% A1c as the threshold for diabetes, and so the Impact Score measured the proportion of participants that had moved below this threshold [3]. The Impact Score was computed using reported mean and standard deviation values and does not reflect any departures from normal distributions. We would expect such departures because participants could have extremely high A1c levels, but not extremely low levels. Therefore, the Impact Score is an approximation rather than a true value. In addition, the effect of a program is more than just the participants who reduced A1c below 6.5%. Any participant able to reduce his or her A1c score would have shown some benefit if this reduction was maintained over time. The purpose of the Impact Score is to help capture the accomplishment of the programs.

These caveats aside, the Impact Scores are revealing. The intervention groups had an estimated 22.84% of participants classified as having A1c values below 6.5%. Thus, almost a quarter of the participants who received the interventions would no longer be considered to have type 2 diabetes. However, at the intervention baseline, 12.73% of participants already had A1c values below 6.5%, so the improvement, while statistically significant, was only 10.11% of the participants. That is, 7.23% of the intervention participants who started with A1c scores of 6.5% or greater achieved a safe level of A1c as a result of the intervention, a result that was significant at the 0.01 level.

Further, the control groups also showed a statistically significant improvement of 2.88%. Therefore, the overall treatment impact (difference in Impact Score between control and intervention groups) was 7.23%. An independent-samples *t*-test revealed that the difference in Impact Scores was significantly greater in the intervention group than the control group, *t*(146) = –4.20, *P* < 0.01. The long-term clinical impact of the successful 7.23% depends on maintaining the A1c reduction over time. The UKPDS [4] report 10 years to be necessary. We find no data about the intensity of DSME needed to maintain A1c reductions over time. 

**Figure 2:**
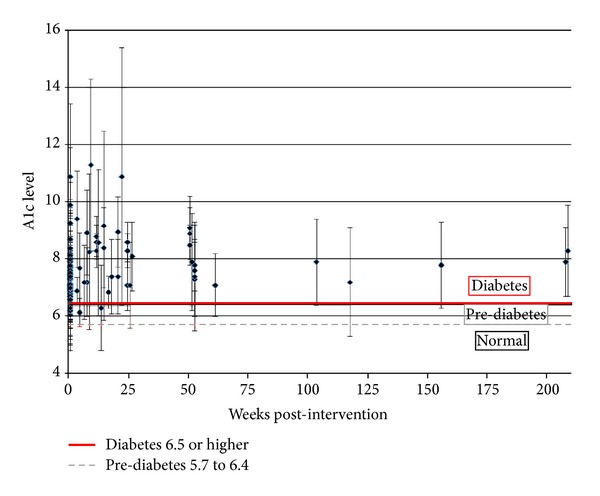
Post-intervention A1c levels.

Finally, we looked at the overall benefit of DSME interventions for participants.  Figure 2 displays the final A1c levels for each study included in the meta-analysis. Even though the interventions were statistically significant, most of the studies failed to achieve the healthy blood glucose level of below 5.7 let alone the pre-diabetes blood glucose level of 6.4.

### 3.2. Intervention Length

Table 3 shows the effects of the duration of the intervention. Longer programs are generally more expensive to conduct and so should result in stronger effects to be considered cost effective. We grouped the studies into three categories: 13 weeks or less, 14–26 weeks, and 27 weeks or more. Each of the three groups showed a statistically significant lower A1c score for the intervention than for the control. The percent difference between the control and the intervention increased monotonically as intervention length increased. The intervention duration of 14–26 weeks resulted in a stronger effect than those lasting 13 weeks or less. The group with the longest duration (27 week or more), showed the highest percent difference (column 9 in Table 3).

A different picture emerged using the mean weighted *d*; the estimated percent below an A1c of 6.5 measure; and the Impact Score. The mean weighted *d* had a larger value for 14–26 weeks than for 27+ weeks. The estimated percent of participants achieving an A1c below 6.5 as well as the Impact Scores were highest for the 14–26 week group. The three measures all declined from 14–26 weeks to 27+ weeks suggesting little reason to extend interventions. These conclusions are tentative because the 27+ weeks category included two studies with sample sizes over 1,000 that had small individual effect sizes and three studies with very small or negative effect sizes.

An analysis of variance of gain scores (intervention mean minus intervention baseline) by intervention length found significant group differences, *F*(2, 71) = 7.60, *P* < 0.01. Follow-up analysis using Fisher's LSD indicated that interventions of 13 weeks or less (*M *difference = −1.51, SD = 1.44) had a significantly greater decrease in A1c levels compared to 14–26 week interventions (*M *= −0.66, SD = 0.68) and interventions that lasted 27 weeks or more (*M* = −0.56, SD = 0.68). These results might suggest that, in addition to being less expensive and easier to administer, shorter interventions can be effective in decreasing A1c levels.

### 3.3. Sustained Effects

Table 4 presents the outcomes for four different delays following the completion of the intervention program: zero delay, 1–13 weeks, 14–26 weeks, and 51+ weeks. None of the studies evaluated A1c between 27 and 51 weeks post-intervention. 

Three of these four durations resulted in significant differences between control and intervention conditions. The 1–13 week condition did not. Most of the studies relied on the immediate post-intervention measurement. The 14–26 weeks condition had the greatest percent difference between intervention and control groups, 8.92%, *t*(9) = 1.83, *P* < 0.05. 

The different analyses varied in their conclusions about sustained A1c reductions although none even approached the 10-year retention intervals associated with health indicators. The Impact Score, at the far right of Table 4, indicates that there is little change in impact over the time intervals used. The Impact Scores for zero delay (7.42), 14–26 weeks (7.87), and 51+ weeks (7.22) were similar, with the highest Impact Score occurring in the 14–26 week group. The Impact Score in the 1–13 week group was the lowest (5.49), partly due to the presence of several studies with very little change between the control and intervention groups. The data in Table 4 are reassuring in that the intervention effects did not quickly disappear. The percent difference column for the zero delay (6.29% improvement, control versus intervention group) may indicate that the intervention was not sufficiently long to take full effect. The percent difference went up for the 1–13 week (8.61%) and the 14–26 week (8.92%) groups, and then fell substantially in the 51+ week condition (5.82%). The mean weighted *d* statistic suggests that the 14–26 week group exhibited the strongest effect. 

Analysis of variance conducted between intervention and control means revealed a marginally significant difference by duration, *F*(3, 70) = 2.21, *P* = 0.09. Tests that were administered 1–13 weeks post intervention (*M *difference = −1.06, SD = 0.75) had a significantly greater decrease in A1c levels compared to tests in the no delay group (*M* = −0.48, SD = 0.50). Furthermore, gain score analysis using one-way analysis of variance by duration revealed significant differences between groups, *F*(3, 70) = 8.97, *P* < 0.001. Both the 1–13 week group (*M* = −1.97, SD= 1.70) and the 14–26 week group (*M* = −2.05, SD= 1.67) reported greater decreases in A1c levels compared to the no delay group (*M *= −0.64, SD = 0.53), and the 14–26 week group also had a larger decrease in A1c levels compared to the 51-plus week group (*M* = −1.16, SD = 0.66). Overall, we found at best weak support for sustained reductions in A1c.

### 3.4. Intervention Methods

We had planned to compare the effectiveness of different intervention strategies but found that 21 studies of the 52 studies used only rules and procedures. Twenty-nine used rules and procedures in conjunction with one or more of the alternative training methods. In contrast, only one study used Affective/Emotional as a single approach. Complex Cognition was used in conjunction with alternative intervention approaches in 18 studies. Affective and Emotional was used in conjunction with alternative intervention approaches in 16 studies. Social and Situational was used in conjunction with other approaches in 10 studies. Only 3 of the 52 programs did not rely on rules and procedures, at least in part. 

The intervention programs that relied entirely on rules and procedures achieved significant reductions in A1c, from 7.71% in the control group to 7.25% (*P* < 0.01). The groups that blended rules and procedures with other types of methods, or relied completely on alternative methods, also achieved a significant (*P* < 0.01) reduction in A1c, from 8.48% to 7.84%. While this is an initial and tentative assessment of methods, the many studies that used more than one method preclude definitive conclusions about method effectiveness. Further, the descriptions of the intervention approaches were often vague and difficult to classify. The synthesis and application of results from complex interventions require particularly careful identification and documentation [74].

#### 3.4.1. Program Presenter

 We examined three classes of intervention presenters: nurse only, nurse in combination with other professional, and no nurse (Table 5). Each was effective in reducing A1c levels, at the *P* < 0.05 levels. The mean weighted *d* was highest for the nurse in combination with others. The percent difference was highest in this condition (8.54%) while the percent difference was lowest in the no-nurse condition, 6.07%. Analysis of variance conducted on the gain scores (intervention mean minus intervention baseline) by program presenter confirmed significant differences between groups, *F*(2, 73) = 7.60, *P* < 0.01. Follow-up analysis using Fisher's LSD indicated that interventions that used a nurse in combination with some other professional (*M difference* = −1.84, SD = 1.66) had a significantly greater reduction in A1c levels compared to nurse only studies (*M* = −0.77, SD = 0.66) and studies that did not use a nurse (*M* = −0.80, SD = 0.65). The results suggest that the addition of a nurse along with other educators or health practitioners might increase the effectiveness of DSME interventions.

## 4. Discussion

### 4.1. Meta-Analysis Outcomes

The present research looks at the daunting challenge of translating medical evidence about Type 2 diabetes self-management into patient decision making, behavioral change, and ultimately blood glucose control. For people with type 2 diabetes, like those with many other chronic conditions, health care providers can prescribe medications, describe optimal dietary patterns, and outline needed life style modifications, but only the patient can implement these critical recommendations. Because adherence depends on patient decisions, we looked at interventions intended to support adherence. We asked: how well are current educational interventions preparing patients to make effective blood glucose control decisions?

First, the good news. Our meta-analysis showed that intervention groups overall showed moderate reductions in A1c from baseline to post-intervention assessment. The average reduction in A1c for the intervention groups was from 8.70 at baseline to 7.61 at the post intervention assessment. The A1c improvements seem fairly robust, 1.09, but must be interpreted in light of the reductions shown by the control participants. The control participants started at the same baseline of 8.70 and reduced it to 8.18, a modest improvement of 0.52. Both experimental and control groups demonstrated a significant (at the 0.01 level) reduction in A1c. 

Improvements in control groups are common and typically attributed to a placebo effect. In the current study, it may also have occurred because some of the studies provided the control group with unspecified “standard training” while the experimental group received innovative training. The intervention group improvement was only 0.57 better than the control group. Nevertheless, it was significantly better (*P* < 0.01). Research suggests that any sustained reduction in HbA1c contributes to patient health [4]. 

Next, the bad news. According to NIH criteria [3], an A1c of 6.5 separates diabetes from pre-diabetes while 5.7 separates pre-diabetes from normal. This means that current DSME intervention outcomes, while laudable, are far from a healthy level. The intervention conditions resulted in small improvements that were sustained over the span of the include studies. The Stratton et al. [4] study of long-term effects found that a 1% reduction, maintained over ten years, conferred clear health benefits. Unfortunately, the intervention effects in the present meta-analysis showed some signs of diminishing over even much briefer study durations. 

 Our findings were more positive than the results of the six previous meta-analyses. Ellis et al. [10] found a reduction of A1c of only 0.32. Gary et al. [11] reported a 0.43 reduction, and Ismail et al. [14] found a 0.32 reduction. We found a reduction of 0.57, compared to the control group. 

 Nevertheless, the Impact Score (the proportion of intervention group participants who moved from a level of 6.5 or above to a level of 6.4 or below, from baseline to post-intervention, in comparison to the control group) was only 7.23%. This is a small achievement in the face of the resources that went into the interventions. Less than 8% of the intervention participants moved below the line for diabetes, compared to the control group. We recognize that the 6.5 level is somewhat arbitrary, but nonetheless it provides a yardstick for assessing program impact. 

In this study, the intervention groups with the shortest durations had significantly greater gain scores. Interventions tended to work at the beginning, but their effects appeared to attenuate over time. This is consistent with Norris et al. [13] that found for studies with follow-ups of a year or more, only two reported sustained A1c reductions. Patients seem to work hard to use rules and procedures at the beginning but have trouble with continued adherence over time. The present meta-analysis seems to replicate greater adherence at the beginning of interventions and later declines. 

This study evaluated DSME interventions. While earlier studies sometimes included people with type 1 diabetes, this study was restricted to people with type 2 diabetes. Unlike some earlier studies, the present sample was restricted to studies using randomized trials. Even with better selection criteria, our outcomes were consistent with earlier research: the benefits of DSME were modest [12, 13]. Further, successful programs are often costly, requiring skilled educators, individualized supervision, and extended time commitments from participants. This makes them impractical to scaleup.

### 4.2. Study Limitations

This meta-analysis has several limitations. First, the 52 studies included were all submitted to and accepted by professional journals. Authors are less likely to submit null findings and editors are less like to accept them. It is, therefore, likely that our outcomes describe more successful interventions. Second, adherent and successful participants are more likely to complete interventions than are less adherent and unsuccessful participants—the problem of attrition rates. Of the 46 studies that reported beginning and end sample size, 10 studies (22%) had attrition rates greater than 20%. Some of the studies in our sample had very high attrition rates (e.g., greater than 40%). Our outcomes are therefore likely to describe more successful studies and the improvements of more successful participants. Taken together, the outcomes are likely to be biased in support of intervention effectiveness.

### 4.3. Rethinking DSME Interventions

The 52 studies we reviewed relied primarily on teaching rules and procedures. A total of 21 programs used rules and procedures exclusively. Only three of the programs did not report using rules and procedures. Our findings show that the rules and procedures approach is effective and its effect is sustained, but modest. The gain score in this category was only a 0.46 reduction compared with that of the control group. The interventions that either blended rules and procedures with other methods, or relied on other methods showed larger improvements over the control group, resulting in a reduction of 0.64. The addition of other strategies, such as complex cognitive or affective interventions might, therefore, serve to enhance the effects of rules and procedures-based methods. Despite the improvement in the intervention groups, the final mean values for both of these conditions were still over 7.0 A1c. 

The interventions had some effect but the effect was not strong enough to help most people avoid the threat of the damages associated with type 2 diabetes over the long term. When people are first diagnosed with type 2 diabetes, we have to send them home with sample menus and lists of foods to avoid. We have to inform them of the dangers of excessive sugar and carbohydrates. We have to convey the procedures for measuring blood glucose levels. Rules and procedures are necessary, but do not appear to be sufficient. 

Lippa et al. [70] conducted Cognitive Task Analysis interviews with people with type 2 diabetes. While rules and procedures were the most common strategy described, this approach is useful but often insufficient. Too often type 2 diabetes patients were burdened with large sets of rules that were poorly understood and difficult to apply. Based on the patterns of successful people, Klein and Lippa [71] concluded that DSME programs should help patients build stronger mental models about the forces they have to juggle—mental models about the tradeoffs between diet, fatigue, exercise, stress, and others. For some, but not all patients, DSME rooted in a cognitive model of system dynamics could supplement the teaching of rules and procedures to help patients with type 2 diabetes become more adaptive and successful. Some patients with type 2 diabetes have used this approach with considerable success.

### 4.4. Recommendations for Future Studies of DSME Programs

We had difficulty in synthesizing different DSME programs because of the lack of standard reporting procedures. Often methodologies and intervention descriptions were too brief and ambiguous to see what actions were actually taken. For example, terms such as “diabetes education program” and “healthy lifestyle” were pervasive and often underspecified. These phrases may involve diet and exercise, but the exact type of education is unknown. Future studies should embed curricula in the text or have links to the material online. 

Also commonly lacking were indicators of the intensity, mechanisms, and presenters of training. Some studies may have achieved better results because of extensive preparation for the intervention facilitators prior to the interview. For example, in Adolfsson et al. [19], the facilitators simulated being diabetes patients for 2 days in order to understand living with diabetes and then underwent workshop training before interacting with a pilot study group. We could not code facilitator preparation because relevant information was rarely provided. It was not always clear whether interventions were done individually or in a group setting. Studies sometimes blurred the lines between who designed the program and who delivered it. They often neglected to identify who presented the intervention to the participants. These variables however are important and should be included in future research reporting.

Program variables are important in evaluating the cost versus effectiveness trade-off. By giving more attention to clarifying their methods, future DSME programs can help to promote progress and contribute to Evidence-Based Medicine. Abraham and Michie [17] demonstrate that standardized definitions of intervention reporting are feasible. We strongly recommend that future research reports detail behavior change techniques and intervention features. This would support efforts to use research result for program development and would serve as a force multiplier for future meta-analyses [17].

## 5. Conclusions

The findings reported in this meta-analysis illustrate the positive but modest gains of existing DSME efforts. There are certainly patients who will have difficultly altering long-term behavioral patterns and others who are simply unwilling to try. Nevertheless, innovative DSME programs that build mental models that help people detect anomalies, identify possible causes, and generate corrective actions hold the possibility of moving more participants to healthy A1c levels. We have come a long way and we have miles to go.

## Figures and Tables

**Table 1:** Description of included studies and interventions.

	Year	*N*	Who delivered	Intervention content	Mode of delivery	Duration of intervention in weeks*	Time before follow-up in weeks*
Adolfsson	2007	88	1, 3b	AE, RP	G	30.33	52
Agurs-Collins	1997	55	4, 0	RP, SS	G, I, S	26	0
Amano	2007	39	0	RP	I	13	0
Anderson-Loftin	2005	65	2n, 4	RP, SS	G, S, T	26	0
Arseneau	1994	40	7	RP, SS	I?	0.57	8.67, 21.67
Barnard	2006	99	1, 4, 6	RP	G, I, T	22	0
Brown	2002	224	3c, 4, 6	RP, SS	G, S	52	0
				1: RP.	1: G, I.		
Campbell	1996	200	3c, 4	2: RP, SS.	2: G, I, S.	2	11, 24
				3: CC, RP, SS.	3: I, T.		
Cheskin	2008	24	4	RP	G, I	86	0
Christian	2008	273	1, 7	AE, RP	I, TECH	52	0
D'Eramo-Melkus	1992	49	0	Group 1: CC, RP. Group 2: CC, RP.	Group 1: G, I. Group 2: G, I.	12, 18	8, 14
Deakin	2006	291	4	AE, CC	G	6	11.33, 54.67
Engel	2006	50	0	AE	GINS, T, TECH	24	0
Faridi	2008	30	3a, 7	RP	TECH	13	0
Fornos	2006	112	3	RP	I, O	56.33	0
Franz	1995	179	2d	RP	I	6	7, 20
Gabbay	2006	332	3c	CC, RP	I, T, TECH	52	0
Gaede	2001	149	1, 3c, 4	AE, RP, SS	G, I, S	197.6	0
Gallegos	2006	45	3c	RP, SS	G, I, T	50	0
Glasgow	1992	97	4, 5, 6	CC, RP, SS	G	13	0
Glasgow	2000	277	2n, 4, 5, 6, 7	RP	O, T, TECH	26	13
Goudswaard	2004	50	3b	RP	I	26	6, 52
Gucciardi	2007	61	3c, 4, 5	CC, RP, SS	G, I	13	0
Janssen	2009	491	1, 3c	RP?	G, I	52	0
Kim & Jeong	2007	51	3c	RP	I, TECH	26	0
Kim & Song	2008	34	3c	RP	TECH	26	0
Ko	2007	308	1, 2n/d, 4, 5	AE, CC, RP, SS	G, S	0.71	25, 51, 103, 155, 207
Krousel-Wood	2008	76	7	RP	TECH	13	0
Kulzer^†^	2007	181	5	1: AE, CC. 2: AE, CC.	G, C: G, I.	13	0, 52
Ligtenberg	1997	51	1, 4	AE, RP	G, I, T	26	0
Lujan	2007	141	6	AE, RP	G, T	26	0
McKibbin	2006	57	6	AE, RP	G	24	0
Ménard	2005	61	0	RP	I, O, T	52	0, 26
O'Kane	2008	184	3a, 4, 6	RP	G	52	0
Pederson	2007	122	0	RP	I, O	26	0
Pibernik-Okanovic	2004	108	4, 5	AE, CC	G	6	7, 20
Piette	2000	248	3c, 7	RP	T, TECH	52	0
Rachmani	2005	110	0	AE, RP	G	208	0, 208
Rosal	2005	25	3c, 4, 6	CC, RP	G, I	10	3, 16
Schwedes	2002	223	1, 3c, 6	CC, RP	G?, I	24	0
Shea	2007	1355	6, 7	CC, RP	TECH	52	0
Sone	2002	1973	3c	RP	I?, T	156	0
Steed	2005	106	3b, 4	CC, RP	G	5	0
Sturt	2008	202	3c	AE, CC, RP	I, O, T	12	14
Sun	2008	146	1, 4	RP	GINS	24	0
Trento	1998	96	1, 5	CC, RP, SS	G, S	52	0
Trento	2002	90	1, 6	CC, RP	G, I+	208	0
Tsujiuchi	2002	26	6	AE	G	17.33	0
Uusitupa	1993	82	1, 3b, 3c, 4	RP	G	65	0, 117
Wattana	2007	147	3c	RP	G, I, O	24	0
Yoo	2008	57	3c, 7	AE	G, I, TECH	13	0
Yoon & Kim	2008	51	6, 7	RP	TECH	52	0

Note. *Studies with multiple intervention lengths or multiple follow-ups are indicated by lengths separated by commas; ^†^A1c values not provided in text—values estimated from a bar graph.

**Who Delivered**: 1 = MD: GPs, or Specialists, 2n = Nurse Certified Diabetes Educator, 2d = Dietician Certified Diabetes Educator, 3a = Nurse Practitioner, 3b = Nurse with Diabetes Specialty, 3c = Nurse (including Nurse Researchers and Educators), 4 = Related Health Professionals: Physical Therapist, Clinical Dietician, 5 = Psycho-social Professionals: Psychologist, Social Worker, Health Counselor, 6 = Other: Professor at Nursing College, Cooking Instructors, Research Assistant, Case Manager, Educationist (MTr), Qi-gong Doctor, 7 = Not a person: Diabetes Manual or Learning-activity-programs, Video, Interactive-telephone-system, 0 = Unlisted, Not Explicit.

**Content**: AE = Affective/Emotion, CC = Complex Cognitive, RP = Rules/Procedures, SS = Social/Situational, ? = Uncertain.

**Mode**: G = Group, I = Individual, T = Telephone, S = Social: Family, Spouse, or Friend Attended, I+ = Individual care given if participant needed additional help, TECH = Technology: Cell Phone Text Messages, Internet, Sensor Placement, Computer Registry, O = Other: Community Resources, Pharmacological, GINS = group or individual not specified, ? = Uncertain.

**Table 2:** Gain score comparison.

	Baseline mean A1c (SD)	Posttreatment mean A1c (SD)	A1c *t*-value (within subjects)	Gain score	Est. % below A1c 6.4 at baseline	Est. % below A1c 6.4 posttreatment	%* t*-value (within subjects)	Impact Score
Control	8.70 (1.48)	8.18 (1.43)	−3.66**	−0.52	11.65%	14.53%	3.15**	2.88
Intervention	8.70 (1.47)	7.61 (1.34)	−8.29**	−1.09	12.73%	22.84%	6.96**	10.11
Significance (between subjects)		*t*(146) = 3.51, *P* < 0.01.		*F*(1, 146) = 7.25, *P* < 0.01				*t*(146) = −4.20, *P* < 0.01.

Note. **P < 0.01. % below A1c 6.4 is an estimate of the percentage of participants in each group who achieved an A1c level below 6.4. Gain Score is A1c change from baseline to posttreatment. Impact Score is the change in estimated percent below A1c 6.4 from baseline to post-intervention.

**Table 3:** Post-intervention A1c levels.

				Control		Intervention									
Length of intervention	*N*	No. of studies	No. of tests	A1c		A1c		Percent difference	*t*-value		Mean weighted *d *	Est. % below A1c 6.4	Control change in est. %	Intervention change in est. %
				*M*	SD	*M*	SD								
13 weeks or less	5,319	17	32	8.22	1.28	7.70	1.29	6.40%		2.13*	0.46	15.68%	4.49%	10.58%	6.09
14–26 weeks	2,247	17	20	8.08	1.58	7.52	1.40	6.89%		1.84*	0.49	21.19%	1.27%	10.74%	9.47
27 weeks or more	6,241	19	22	8.20	1.52	7.56	1.37	7.79%		2.03*	0.23	19.86%	2.02%	8.84%	6.82

Note. *P < 0.05. One article [16] included two intervention groups that were 12 weeks and 18 weeks and is therefore counted twice in the number of studies column. % below A1c 6.4 is an estimate of the percentage of participants in the intervention groups who achieved an A1c level below 6.4. Change in est. % is the change in estimated percentage of participants' baseline to post-treatment. Difference in change in est. % is the difference between the control and intervention groups.

**Table 4:** Mean outcome A1c levels for control and intervention groups, by delay from end of intervention to time of test.

				Control		Intervention								
Length of delay	*N *	No. of studies	No. of tests	A1c		A1c		Percent difference	*t*-value	Mean weighted *d *	Est. % below A1C 6.4	Control change in est. %	Intervention change in est. %	Impact Score
				*M *	SD	*M *	SD							
No delay	8,729	39	43	7.92	1.45	7.42	1.29	6.29%	2.31*	0.29	21.46%	3.16%	10.58%	7.42
1–13 weeks	1,291	7	9	8.38	1.36	7.66	1.15	8.61%	1.65^†^	0.12	13.66%	1.89%	7.38%	5.49
14–26 weeks	1,572	9	11	9.07	1.54	8.26	1.56	8.92%	1.83*	1.02	11.66%	1.08%	8.95%	7.87
51 weeks or more	2,215	8	11	8.13	1.30	7.65	1.47	5.82%	2.50*	0.32	19.76%	4.41%	11.63%	7.22

Note. *P < 0.05. ^†^ *P* < 0.10. % Below A1c 6.4 is an estimate of the percentage of participants in the intervention groups who achieved an A1c level below 6.4. Change in est. % is the change in estimated percentage of participants from baseline to post-treatment. Difference in change in est. % is the difference between the control and intervention groups.

**Table 5:** Mean Outcome A1c levels for control and intervention groups, by type of professional who delivered intervention.

				Control		Intervention				
Who delivered intervention	*N *	No. of studies	No. of tests	A1c		A1c		Percent difference	*t*-value	Mean weighted *d *
				*M *	SD	*M *	SD			
Nurse only	2,996	9	10	8.18	1.39	7.58	1.34	7.24%	2.32*	0.17
Nurse in combination with others	3,275	14	21	8.38	1.17	7.67	1.01	8.54%	2.01*	0.59
No nurse	7,536	29	43	8.08	1.57	7.59	1.5	6.07%	2.32*	0.34

Note. *P < 0.05.
